# Synthesis of Bis(dodecylammonium) Tetrachlorocuprate Using Ball Milling for Thermal Energy Storage

**DOI:** 10.1021/acssuschemeng.4c08677

**Published:** 2025-03-26

**Authors:** Rebeca Salgado-Pizarro, Jofre Mañosa, Camila Barreneche, Ana Inés Fernández

**Affiliations:** Departament de Ciència de Materials i Química Física, Secció de Ciència de Materials, Facultat de Química, 16724 Universitat de Barcelona, C/Martí i Franquès 1-11, Barcelona 08028, Spain

**Keywords:** energy storage materials (ESM), thermal energy storage (TES), ball milling, green chemistry, sustainable evaluation

## Abstract

Layered hybrid halometallates are highlighted for their adaptable thermal, electrical, and optical properties by modifying the alkylammonium or metal constituents. However, the conventional synthesis procedures of these materials present some sustainability and scalability issues. To tackle these issues, mechanochemistry is a promising alternative synthesis which uses mechanical energy and can reduce the solvent content required. Mechanochemical synthesis has proven to be an effective synthesis route for various perovskite structures, but research on bis­(alkylammonium) tetrahalometallates is limited. Here, we explore the feasibility of synthesizing bis­(alkylammonium) tetrahalometallates through ball milling and reducing solvent usage. Crystal and molecular results confirmed the successful synthesis with minimal impurities, <5 wt %. The bis­(alkylammonium) tetrahalometallates obtained through ball milling presented comparable enthalpy and specific heat values to those obtained through traditional synthesis routes. Moreover, the ball milling synthesis consumed significantly less energy and solvent and led to higher reaction yield than the traditional synthesis methods, thereby enhancing the sustainability of the process. Overall, the results validate the synthesis through ball milling as a viable and environmentally efficient method for bis­(alkylammonium) tetrahalometallates.

## Graphical abstract

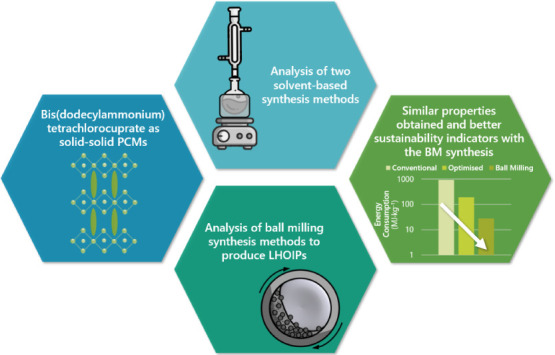

## 1 Introduction

The layered hybrid halometallates have recently gained attention in different fields, including thermal, barocaloric, and magnetocaloric energy storage, due to their remarkable physical and chemical properties.^1–5^ Furthermore, these materials are highlighted by their first-order phase transition in solid-state and present a high degree of flexibility in customization, as their properties can be tailored by modifying the alkylammonium or the metal employed.^6^

The versatility of this family of materials resides in their unique perovskite structural arrangement, which consists of an octahedral organization of the metal atom surrounded by the halide ion and the organic cation (protonated alkylamine) in the interstitial sites. This results in a layered structure with a high degree of customization in terms of thermal, electronic, and optical properties.^6–10^

Typically, the synthesis protocol to prepare these compounds consists of reacting the stoichiometric amount of alkylamine, metal chloride, and hydrochloric acid in absolute ethanol or methanol under constant refluxing for four h and then cooling down the solution to allow the precipitation and growth of the desired crystals. Finally, the crystals are filtered and recrystallized several times.^11–13^ However, conventional methods present sustainability issues because they use and dispose of large amounts of solvent, are energy-consuming since long refluxing times are required, and present scalability issues.

Recently, mechanochemistry has emerged as an alternative synthesis route for organic and organometallic compounds that is simpler, more sustainable, and more scalable than traditional synthesis.^14–18^ The base of mechanochemistry is the use of mechanical energy (compression, shear, or friction) to induce a chemical transformation.^19^ Mechanochemistry is widely used as an alloying technique to produce ternary oxides, nanoparticles, halides, sulfides, or nitrides.^20–23^

Different studies report that mechanochemical synthesis is a reliable and solvent-free route to produce 3D, 2D, and 1D advanced hybrid perovskites.^24–29^ There is a large variety of mechanochemical synthesis methods, ranging from mechanical roller and planetary ball mills to grinding with an agate mortar. However, the type of grinding source used can result in variations in the final product. Additionally, the jar and ball materials can play an important role. It is essential to ensure compatibility with the reactants being used and the energy required to perform the synthesis, as different impact energies will be generated based on the materials used.^18,30–32^

The literature describes several protocols for producing hybrid perovskites. Jodlowski et al. proposed a solvent-free protocol for producing 3D, 2D, and 1D advanced hybrid perovskites using a planetary ball mill, with a 125-mL stainless steel jar and 16 stainless steel balls, 10 mm in diameter, using the stoichiometric amount of reactants at 350 rpm for 10 min.^24^ Baek et al. suggested a similar procedure for synthesizing Cs–Pb–Br perovskites, which involved employing a mechanical roller. They used the stoichiometric amount of reactants in an alumina jar and different amounts of stainless-steel balls at 350 rpm for 48 h.^25^ However, no research has been found in the literature regarding the mechanochemical synthesis of bis­(alkylammonium) tetrahalometallates.

The present study aims to assess the possibility of using ball milling to synthesize bis­(dodecylammonium) tetrachlorocuprate (CuC_12_) and evaluate the feasibility of reducing the solvent content compared to traditional synthesis methods. The main thermophysical properties, material purity, and impurity content were evaluated, as well as the main thermal properties to use this material as a solid–solid phase change material. Additionally, we investigated a series of key environmental parameters, comparing the conventional and ball milling synthesis methodologies to find the most efficient and sustainable method to produce bis­(alkylammonium) tetrahalometallates.

## 2 Experimental Procedure

### 2.1 Materials

The following were the raw materials employed to synthesize the bis­(alkylammonium) tetrahalometallates (CuC_12_). *N*-dodecylamine 98%, C_12_H_27_N (CAS No. 124-22-1), was purchased from Acros Organics. Copper­(II) chloride dihydrate, CuCl_2_·2H_2_O (CAS No. 10125-13-0), was purchased from VWR International. Hydrochloric acid 37%, HCl (CAS No. 7647-01-0), was purchased from Labbox, and anhydrous methanol (max 0.003% H_2_O) (CAS No. 67-56-1) was purchased from Merck Group.

### 2.2 Synthesis Procedures

Eight samples were evaluated: two were obtained by traditional synthesis routes and six by ball milling.

For the synthesis through ball milling (BM), 6.13 g of C_12_H_27_N_(s)_, 3.04 g of CuCl_2_·2H_2_O_(s)_, and 3 mL of HCl_(aq.)_ were used. All of the reagents required were added to a 250-mL alumina grinding jar with 27 alumina balls of 10 mm diameter. The solvent content employed in each sample was modified in order to evaluate its effect on the synthesis, in which the solvent-to-mass ratio was altered from 7.6 to 0.5, where 7.6 is the same ratio employed in the traditional methods. The nomenclature for each sample correlates with the solvent-to-mass ratio (BM_7.6, BM_5.7, BM_3.8, BM_2.0, BM_1.0, and BM_0.5).

All of the samples were obtained following the same procedure: 12 min of grinding at 300 rpm using a RETSCH PM100 planetary ball mill. After being milled, the jars were opened, and the product was transferred to Petri dishes. Any remaining product in the jar was extracted by adding 15 mL of the solvent. The product was initially dried for 7 days at room temperature, followed by 30 min at 333 K. The final dried product was ground with an agate mortar for further testing. The yield of the ball milling synthesis ranged from 81% to 97% relative to the theoretical amount, with the highest yield obtained for the BM_5.7.

For the traditional synthesis, two different routes were considered in this study: the conventional synthesis (Conv.), which is well described in the bibliography, and an optimized synthesis (Optim.), which is presented in previous work.^11–13,33^ For both synthesis routes, the stoichiometric molar amount of the reagents, as presented in eq 1, was added into a round-bottomed flask with 45 mL of anhydrous methanol. While the products were under constant stirring, HCl, dissolved in 5 mL of anhydrous methanol, was added dropwise through the condenser. The solution was refluxed at 337 K under continuous stirring for 4 h.

1 $$2\left(CH_{3}{\left(CH_{2}\right)}_{11}NH_{2}\right)+2HCl+CuCl_{2}\to {\left(CH_{3}{\left(CH_{2}\right)}_{11}NH_{3}\right)}_{2}CuCl_{4}$$

For the Optim. synthesis, after the reflux step, the solution was poured into a Petri dish and dried in a desiccator for 7 days. For the Conv. synthesis, after refluxing, three successive recrystallizations in anhydrous methanol were performed by adding ∼15 mL of solvent to dissolve the product, and the solution was refluxed at 337 K for 45 min. Subsequently, the final solid was dried in a desiccator for 7 days at room temperature.

In both cases, a final drying step was performed, which consisted of heating the product at 333 K for 30 min. The final dried products were ground with an agate mortar for further analysis. The yield of the Conv. synthesis was 66%, and the Optim. synthesis achieved an 88% yield.

### 2.3 Characterization

#### 2.3.1 Crystal Structure

The structure of the samples was determined by X-ray powder diffraction (XRD) with a PANalytical X’Pert PRO MPD diffractometer of 240 mm radius, in a configuration of convergent beam with a focalizing mirror and a transmission geometry. The flat samples were sandwiched between low-absorbing films of polyester mylar of 3.6 μm of thickness. The measurements were performed with Cu Kα radiation (λ= 1.5418 Å), a voltage of 45 kV, and a tube current of 40 mA. The measurements were obtained in continuous scan mode, with a 2θ range from 1° to 40°, step size of 0.0263° 2θ, and a measuring time of 300 s per step. The quantification of the crystalline phases was performed through Rietveld refinement by using TOPAS software.

#### 2.3.2 Elemental Analysis

Inductively coupled plasma mass spectrometry (ICP-MS) was used to determine the aluminum content in the samples and assess potential contamination due to abrasion. The measurements were performed using a NexION 2000 (PerkinElmer) instrument after microwave digestion of the samples with HNO_3_ at 240 °C, in duplicate.

#### 2.3.3 Molecular Structure

Fourier transform infrared spectroscopy (FTIR) combined with attenuated total reflectance (ATR) (PerkinElmer Spectrum Two) was used to determine the molecular structures and analyze the chemical changes that occurred during the synthesis. Proton nuclear magnetic resonance (^1^H NMR) spectra were recorded at 400 MHz in methanol-d4 (CD_3_OD) using a Bruker BioSpin GmbH 400 MHz NMR spectrometer to confirm protonation of the amine during the synthesis.

#### 2.3.4 Morphology

A scanning electron microscope (SEM) was used to examine the morphology of the synthesized structures, using an XTE 325/D8395, Quanta 200 FEI. Several representative flakes were selected before grinding the samples, which were attached to a carbon adhesive and coated with carbon to acquire SEM images and perform energy-dispersive scattering (EDS) analysis of the surface.

#### 2.3.5 Thermophysical Analysis

The thermal decomposition of the samples was analyzed using thermogravimetric analysis (TGA) under a synthetic air atmosphere (50 mL·min^–1^) using a TGA 550 (TA Instruments) from 300 to 900 K and with a heating rate of 10 K·min^–1^. The phase transition of the samples was investigated by using differential scanning calorimetry (DSC) (Mettler Toledo DSC 822e Star 3+). The phase transition temperature (Tt) and enthalpy (ΔH) were evaluated between 303 and 353 K, with a heating rate of 1 K·min^–1^ and a nitrogen flow of 50 mL·min^–1^. Each analysis was repeated three times, with the first being discarded due to contact issues between the sample and the crucible. Moreover, the DSC was used to determine the specific heat (Cp) at 290 to 380 K, with 10 K intervals, using the approach established by Ferrer et al.^34^ In this approach, the sapphire is analyzed as an internal standard, and the sample is analyzed in successive isothermal segments, without heating ramps and with a temperature difference between the isothermal steps of 1 K. Heat flow peaks represent the heat difference between the isothermal steps. By integrating these peaks on the sapphire and the sample, the *C*_p_ value is calculated using eqs 2 and 3.

2 $$A=C_{p}\cdot \beta$$

3 $$C_{pm}=\frac{C_{ps}\cdot A_{m}}{A_{s}}$$

### 2.4 Environmental Assessment

The environmental assessment was performed through key indicators commonly used to evaluate “greener” procedures.^35^ The selected indicators were as follows : the real atom economy (RAE) considers the stoichiometry and the reaction yield, and the value should ideally be near 100.^36^ The RAE indicator identifies the synthetic efficiency by the maximum number of atoms present as a reactant shall be incorporated into the reaction products, taking into account the yield of the synthesis.^37^ The environmental factor (E-factor) evaluates the waste generated during the reaction, considering the reagents, solvent losses, and additives used during the synthesis process concerning the mass of the product obtained, where the optimal value should be close to 0.^38^ The process mass intensity (PMI) also assesses the waste generated during the synthesis process by considering the total mass processed, with respect to the mass of the product. This indicator can also be obtained by adding 1 to the E-factor, as 1 is the ideal result of the PMI indicator.^39^ The EcoScale is a qualitative metric commonly used in organic synthesis. It considers the reaction yield, the cost of the reagents, safety issues, reaction parameters (such as temperature and time), and purification steps. The main idea is to designate penalty points for each parameter, and the EcoScale is the difference between 100 and the sum of all the penalty points.^40^ The energy consumption (MJ·kg^–1^) of each process was also included, as it can be directly related to the environmental footprint of the process and is an essential factor that needs to be considered for the scale-up and the industrialization of the process. Here, we determined the energy consumed to produce the desired product using an energy meter.^41^

## 3 Results and Discussion

### 3.1 Crystal Structure

The X-ray diffractograms are presented in Figure 1. The low-angle peaks at 6°, 9°, 12°,···, are characteristic of a two-dimensional layered crystalline structure, where each peak is related to the reflection on the (00l) planes, which are strongly oriented along the *c*-axis.^13,42–44^ The main peaks of the CuC_12_ structure were found in the BM samples, validating that synthesis through ball milling is suitable for obtaining this material. Furthermore, the products obtained from the ball milling process of the sample with the lowest solvent content, BM_0.5, were analyzed using XRD immediately after milling, without any drying step. The results revealed the principal peaks of the CuC_12_ structure, confirming that CuC_12_ formed during the ball milling process; see Figure S1. However, some impurities were detected in the diffractograms of the BM samples; see Figure 1. The impurities detected were unreacted CuCl_2_·2H_2_O at 16.14°, 21.95°, and 33.98° 2θ (marked with rhombi), and dodecylamine hydrochloride (C_12_H_28_NCl) presents a stackable two-dimensional structure (marked with squares) in Figure 1.^45^ To determine the purity of the BM samples and quantify the impurity content, the Rietveld refinement was employed with the TOPAS software using the structures 2305992 from CCDC,^46^ 955864 from CCDC,^45^ and 1568587 from COD.^47^ The refinement results are presented in Table 1. All the refinements achieved a proper weighted profile R-factor (Rwp). Moreover, all the samples evaluated presented adequate purity (>95 wt %), validating the suitability of ball milling and the possibility of reducing the solvent content to obtain CuC_12_. Additionally, the aluminum content was determined using ICP-MS in the samples with the highest and lowest solvent content, BM_7.6 and BM_0.5, obtaining 515.2 and 30.6 ppm, respectively. These results confirm the adequacy of the proposed method and indicate minimal abrasion between the alumina balls and the jar.

**Figure 1:**
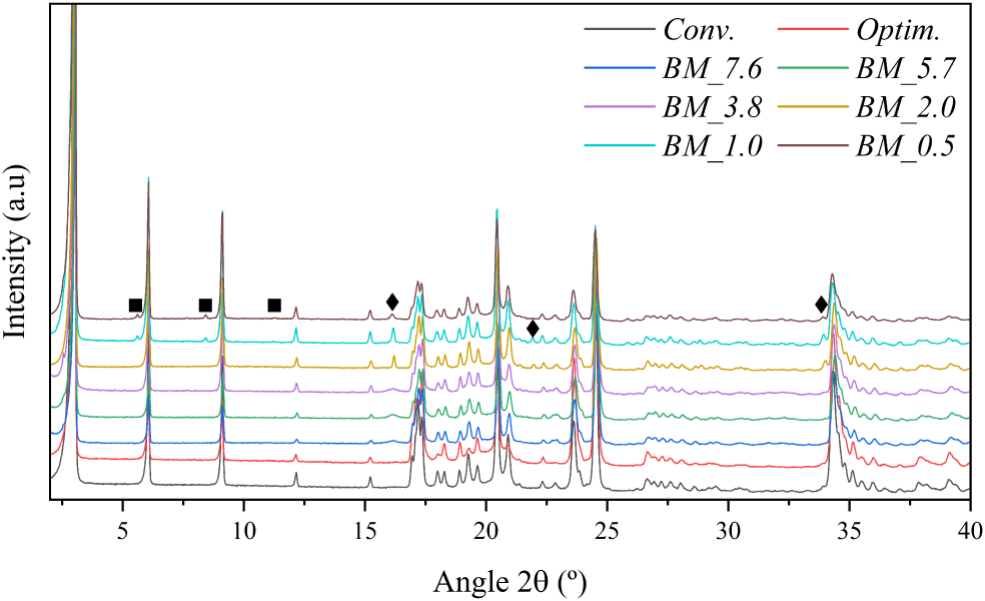
XRD diffractograms of CuC_12_ obtained through BM, compared to traditional synthesis methods.

**Table 1:** Rietveld Refinement of the CuC_12_ Samples Obtained Through BM

Samples	CuCl_2_·2H_2_O (wt %)	C_12_H_28_NCl (wt %)	CuC_12_ (wt %)	*R*_wp_
***BM_7.6***	1.33	0.59	98.08	12.629
***BM_5.7***	0.95	2.13	96.31	12.747
***BM_3.8***	1.3	3.4	95.31	11.870
***BM_2.0***	2.59	-	97.41	12.589
***BM_1.0***	2.64	2.03	95.33	12.980
***BM_0.5***	1.42	2.57	96.01	12.681

### 3.2 Chemical Structure

Regarding the organic part of the structure, the FTIR results show that all the samples present the same functional groups, as they have similar FTIR spectral profiles; see Figure 2. The amine functional group was detected through the N–H stretching vibration band at 3500 to 3250 cm^–1^ and the symmetric and antisymmetric deformation bands of R-NH_3_^+^ at 1591, 1491, and 1480 cm^–1^, respectively. Additionally, the C–N stretching and the R-NH_3_^+^ wagging bands were detected at 1215 and 769 cm^–1^, respectively.^1,42^ For the carbon chain, the R-CH_3_ and R-CH_2_-R asymmetric and symmetric bands were detected at 2955, 2871, 2917, and 2849 cm^–1^, respectively. The bending R-CH_2_-R band at 1472 and 1463 cm^–1^, the symmetric bending of R-CH_3_ at 1377 cm^–1^, rocking R-CH_2_-R at 728 and 720 cm^–1^, and the terminal C–C stretching at 891 cm^–1^ were detected.^1,42,48,49^ Moreover, the R-CH_2_-R bending and the R-CH_2_-R rocking bands are nonlocalized vibration modes, which agree with the literature.^33,48^ Additionally, the bands between 3500 and 3250 cm^–1^, associated with amine protonation (RNH^+^), seem slightly more intense in the BM samples, suggesting a difference in the material’s chemical environment compared to the conventional synthesis. However, this higher intensity may not be directly linked to an increased concentration of protonated amine. Instead, this could be attributed to the presence of dodecylamine hydrochloride that remains unincorporated into the layered perovskite structure. Moreover, at 1628 cm^–1^, an extra peak is detected in the BM_7.6, BM_5.7, and BM_3.8, related to unreacted amine.

**Figure 2:**
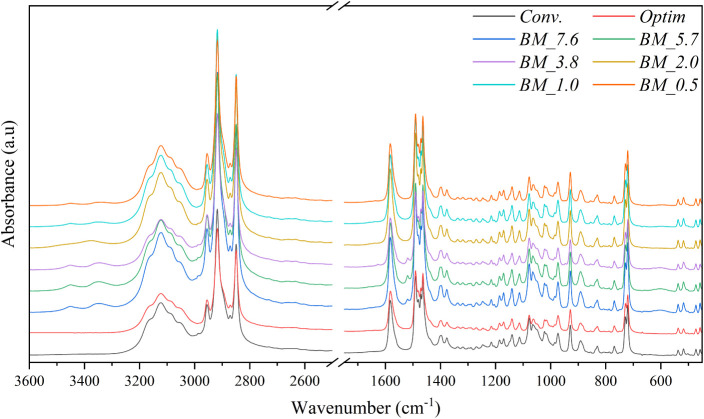
FTIR spectra of CuC_12_ obtained through ball milling and traditional synthesis methods.

The ^1^H NMR spectra of the different components, shown in Figure 3, exhibit peaks corresponding to the organic chain of the components. In all cases, the main peaks of the alkylamine chain are clearly detected, along with the protonation shifts around 7.5 ppm, confirming successful amine protonation during ball-milling synthesis. Additionally, residual water and CH_2_DOD from the solvent were detected and assigned to the spectra. The close similarity of the ^1^H NMR between the samples synthesized by ball milling and the sample obtained by a classical methodology (Optim.) further validates the protonation process. Due to the paramagnetic nature of the (CuCl_4_)^2–^ moieties, the NMR signals are not sufficiently clear to enable quantification of the ^1^H NMR.

**Figure 3:**
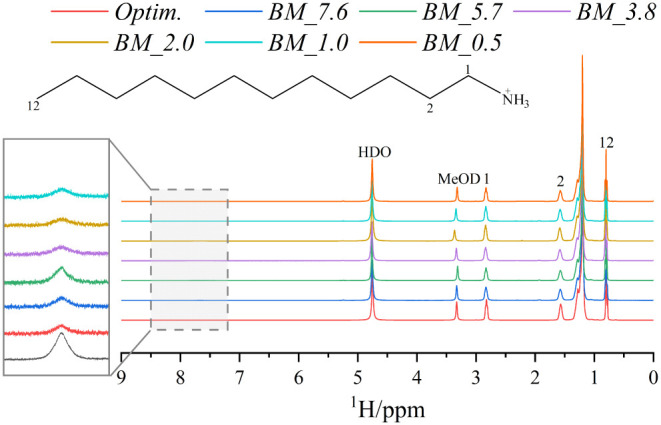
^1^H NMR spectra of the CuC_12_ synthesized by ball milling and traditional synthesis methods.

### 3.3 Morphology Study

The morphology of the samples was evaluated by using a scanning electron microscope (SEM), as shown in Figure 4. Differences between the two synthesis routes are observed. The samples obtained by traditional synthesis routes (Conv. and Optim.) show a faceted crystal plate morphology and larger plates. In contrast, the BM samples exhibit slightly rounded plate edges due to mechanical action during milling, mainly in BM_2.0, BM_1.0 and BM_0.5, where the solvent content was drastically reduced, leading to a reduction in crystal sizes. However, the overall shape remains consistent across all samples, showing a crystal plate morphology.

**Figure 4:**
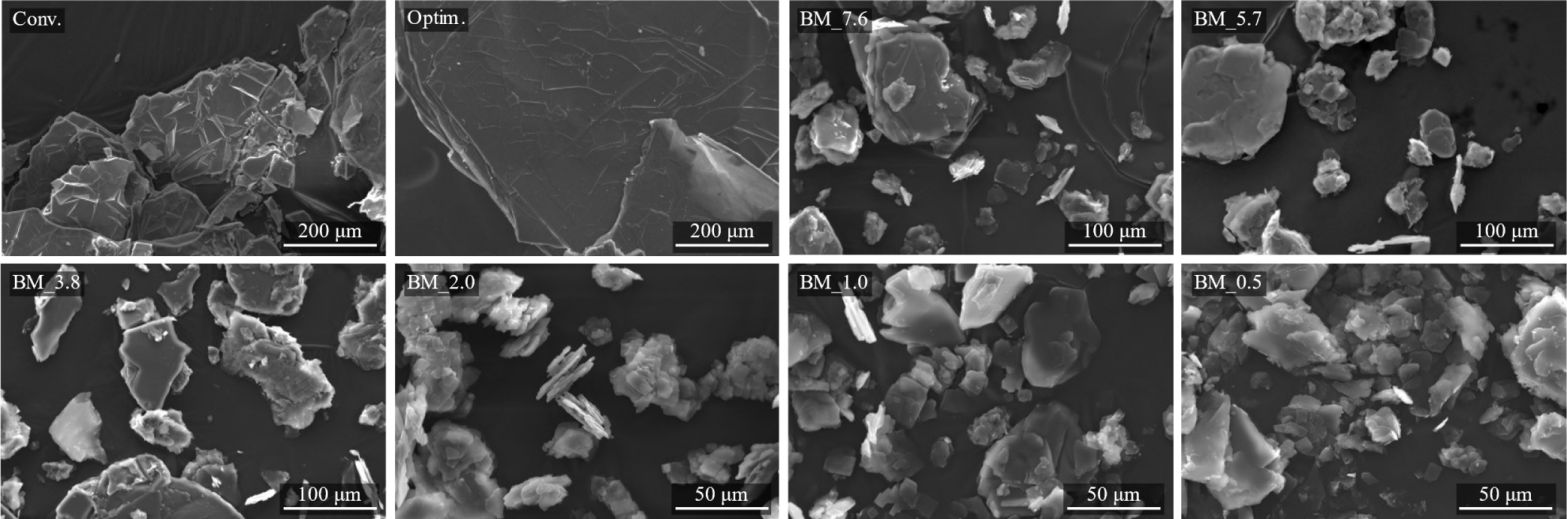
SEM images of the Conv., Optim., and BM samples.

Despite variations in the amount of solvent used, there were no notable differences in the morphology of the BM samples, except for some variations in particle size. The larger crystal growth observed in the conventionally synthesized samples (Conv. and Optim.) compared to those prepared through the BM route can be attributed to the fact that the conventional samples were still warm when left to dry, potentially promoting greater crystal growth. In contrast, the BM route, which involves milling, limits this growth due to the lower temperature of the samples during the drying phase.

### 3.4 Thermophysical Analysis

As described in previous studies, CuC_12_ presents two main transitions in the solid state. The first transition occurs around 329 K with an enthalpy between 60 and 70 J·g^–1,^ and the second transition occurs around 336 K with an enthalpy of 10–12 J·g^–1^.^33^ The mechanisms responsible for the two transitions reside in the reorganization of the carbon chain.^50^ However, the perovskite layer is responsible for the material transitioning into a solid state rather than a solid–liquid state, as the ammonium group is attached to the perovskite layer, blocking its free movement.^51^ Figure 5 displays the transition enthalpy (bars) and temperature (rhombuses). None of the samples presented significant differences, meaning that we obtained the desired material and neither the synthesis route nor the solvent content affected the thermal properties. BM_1.0 presents relatively lower enthalpy values, mainly in the first transition.

**Figure 5:**
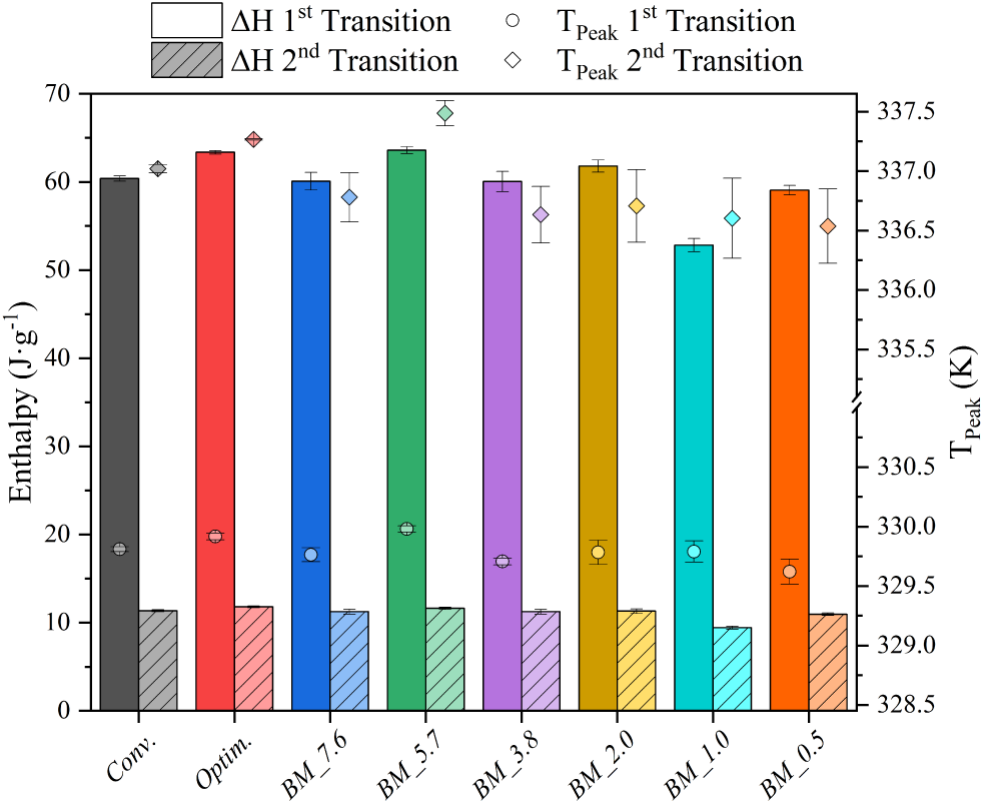
Enthalpy and transition temperature of CuC_12_ samples obtained by ball milling and traditional synthesis methods.

Furthermore, samples BM_1.0 and BM_0.5 present additional peaks, as shown in Figure 6. These extra peaks could be due to the presence of dodecylamine hydrochloride found in the XRD results. Regarding the other samples, no discrepancies were found from the traditional methods, despite small shifts in the peak temperature, which may be caused by the different organizations of the carbon chains.

**Figure 6:**
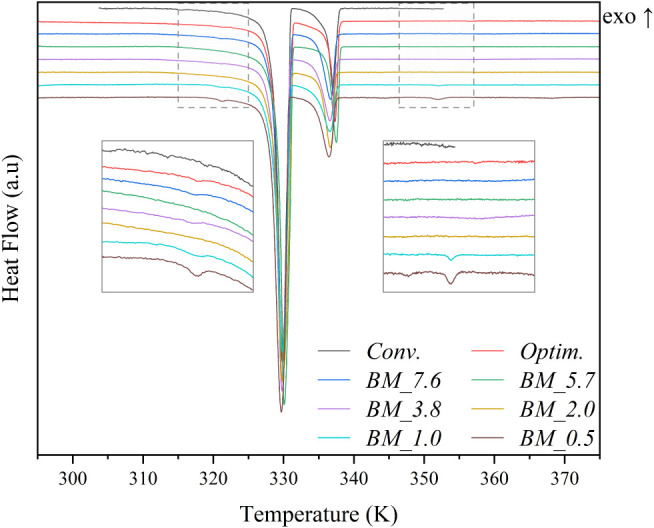
Heat flow curves of Conv., Optim., and BM samples.

Figure 7 displays specific heat values as a function of the temperature of the samples selected to be analyzed and the heat flow curve of the Conv. sample. As a common trend across all the samples, the specific heat values exhibit an increase as the temperature approaches the polymorphic transition temperature. Following the phase transition, the specific heat values stabilize up to 380 K as the system has reached a new thermodynamic equilibrium. The highest specific heat is observed in the Optim. and BM_2.0 samples, while Conv. and BM_5.7 present the lowest values. Improved crystallinity often leads to more organized lattice structures, facilitating efficient heat transfer and increased capacity for thermal energy storage.^52–54^ Theoretically, we expect that with traditional methods, we will obtain better crystallinity and larger crystal sizes compared to the ball-milled samples. Contrary to this hypothesis, Conv. samples present the lowest values of specific heat. However, the value differences are minimal and could be attributed to equipment error.

**Figure 7:**
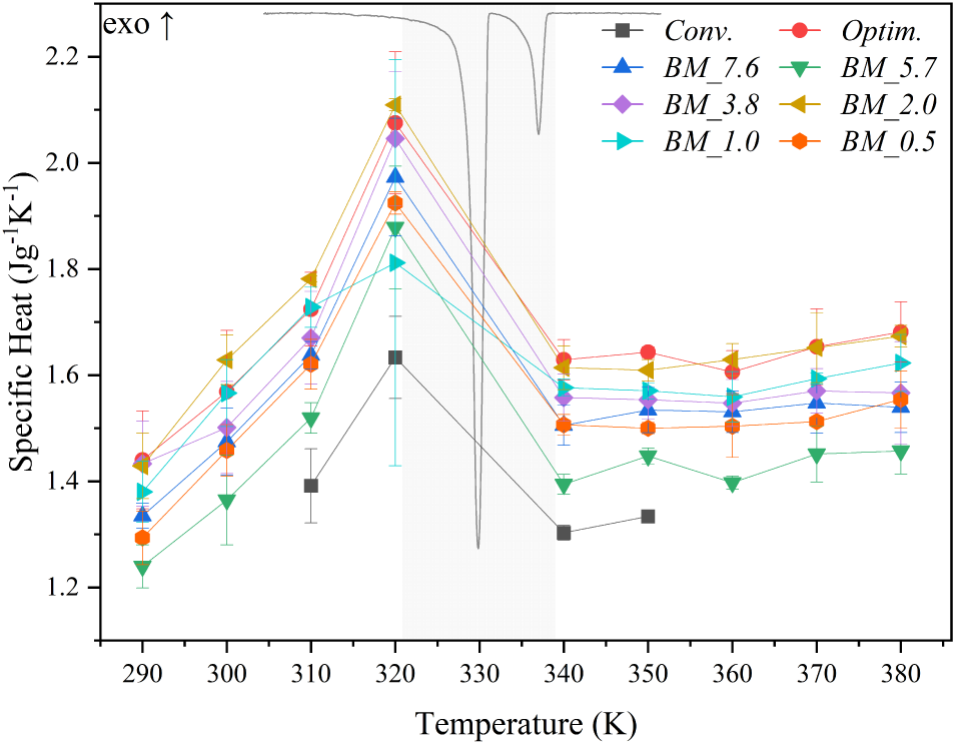
Specific heat results of Conv., Optim., and BM samples.

In the thermogravimetric analysis presented in Figure 8, it is observed that all of the samples show a similar profile, composed mainly of four thermal decomposition steps. The first step (I) occurs up to 420 K due to the detachment of some water content, which can be observed in the samples BM_5.7, BM_2.0, and BM_1.0. The second step (II), up to 595 K, corresponds to the degradation of the carbon chain of the component, showing a weight loss of around 56 wt %.^55,56^ The third step (III) happens up to 700 K, which is related to the decomposition of the degradation products of the alkylamine, presenting a weight loss of around 8 wt %.^56^ The final step (IV), up to 900 K, involves decomposing the residual metal complex, releasing the chloride content, with a weight loss of about 29 wt %.^57^ Additional decomposition steps (A) can be found at 450–500 K in the BM samples due to the decomposition of some unreacted dodecylamine hydrochloride, also detected in the XRD and DSC analyses. A summary of the different decomposition step values is presented in Table 2. The BM samples displayed very similar curves, confirming that the reduction of the solvent content did not significantly alter the structure of CuC_12_ obtained.

**Figure 8:**
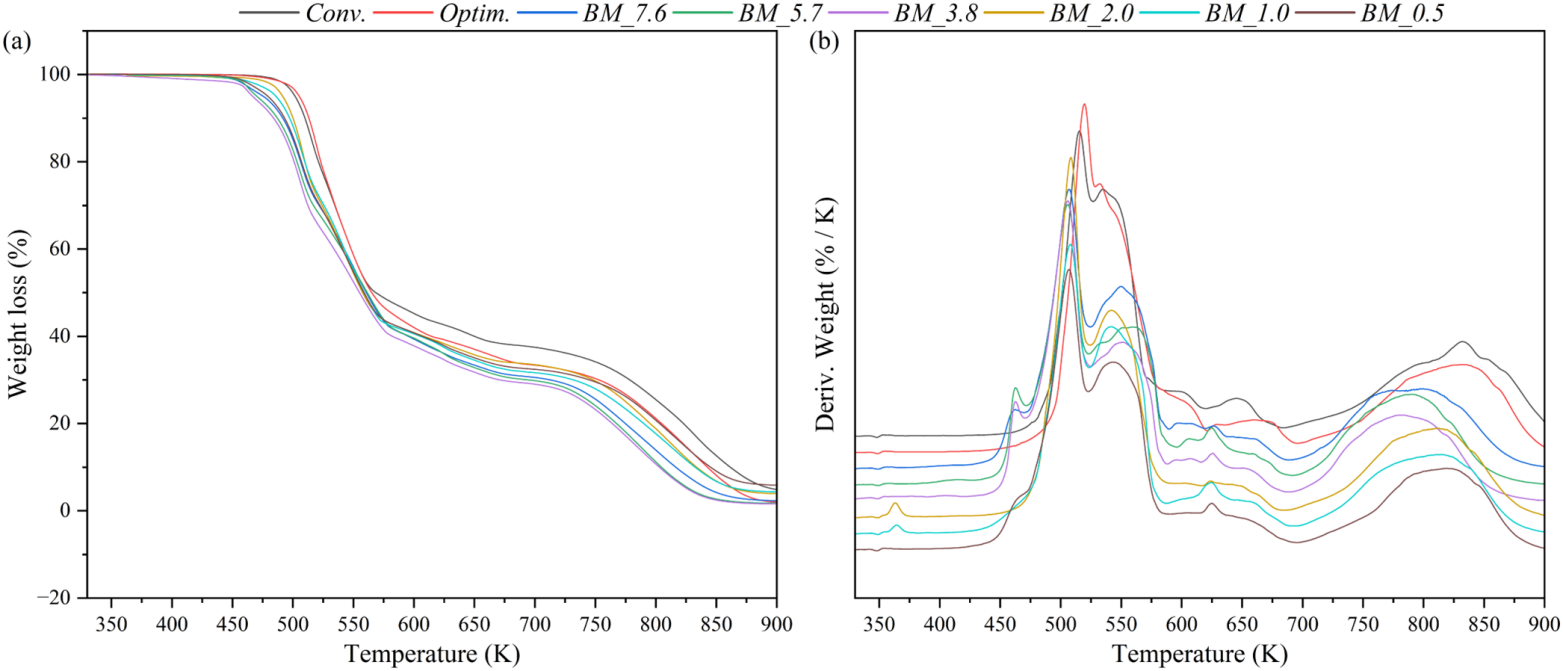
Thermograms (a) weight loss and (b) weight derivative curve as a function of the temperature of CuC_12_ samples obtained by ball milling and traditional synthesis methods.

**Table 2:** Summary of Weight Loss at Each Step for All Analysed Samples

	Weight Loss Steps (%)				
Sample	I	A	II	III	IV
***Conv.***	-	-	57.13	4.95	33.42
***Optim.***	-	-	60.59	5.89	31.64
***BM_7.6***	-	4.54	54.69	9.89	28.62
***BM_5.7***	0.55	5.00	54.56	9.84	28.40
***BM_3.8***	-	6.56	54.20	9.83	27.93
***BM_2.0***	0.42	-	58.95	6.82	30.02
***BM_1.0***	0.25	2.40	55.49	9.98	27.44
***BM_0.5***	-	3.86	54.39	9.29	26.67

### 3.5 Sustainability Evaluation

The efficiency and the environmental impact of the different synthesis methods studied in this work are presented in Table 3 through common key indicators, with the complete data provided in the Supporting Information. The RAE evaluates the synthesis efficiency by calculating the coefficient of the actual weight of the desired product by the total weight of all the materials used in the process. Hence, values nearing 100% indicate effective synthesis. The lowest value (2%) corresponds to the Conv. synthesis. The sample that presents the highest value (49%) is BM_0.5, as the lowest content of the solvent was used. This indicates that the Conv. synthesis is highly inefficient, while the process involving BM_0.5 is very efficient, showing that a decrease in the solvent content led to higher RAE, ranging from 12% in BM_7.6 to 49% in BM_0.5.

**Table 3:** Summary of the Key Sustainable Indicators for Each Synthesis Method

Indicator\Synthesis Method	*Conv.*	*Optim.*	*BM_7.6*	*BM_5.7*	*BM_3.8*	*BM_2.0*	*BM_1.0*	*BM_0.5*
Real atom economy (%)	2	11	12	17	19	30	45	49
Environmental factor	41.67	7.51	6.82	4.45	3.54	1.79	0.80	0.45
Process mass intensity	45.68	8.89	8.39	5.82	5.16	3.36	2.20	2.03
EcoScale (%)	35	49	51	58	50	51	57	51
Energy consumption (MJ·kg^–1^)	937.48	193.63	28.49	24.78	29.54	28.47	25.39	28.64

Regarding the sustainability factors, the E-factor is a critical indicator that measures the waste generated—in our case, the solvent used per product unit. The Conv. method presents the highest values, 41.67%, compared to the alternative methods, where the lowest values correspond to BM_1.0 and BM_0.5 as less solvent is used, significantly reducing the impact of the waste generated by the solvent. However, the Optim. and BM_7.6 have similar values since similar solvent content was used and present similar reaction yields. A similar phenomenon occurs with the PMI, which relates to the raw material used per product unit. Hence, the lower the PMI, the higher the efficiency of the resources used. The EcoScale is a qualitative evaluation that tries to elucidate the environmental performance of a method by incorporating penalty points for different parameters, and the ideal method is the one that presents low penalties, achieving a score near 100. In our study, the BM samples present higher values than the traditional methods.

Finally, energy consumption can be used to evaluate the energy efficiency of each method. Remarkably, the ball milling method consumes approximately seven times less energy than the Optim. method and over 30 times less than the Conv. method. This assessment demonstrates that synthesis through ball milling is a potential alternative synthesis route with superior sustainability results compared with traditional methodologies. Furthermore, a reduction in the solvent content caused a reduction in the waste generated, also improving the sustainability of the process.

## 4 Conclusions

In this first study, we aimed to evaluate the feasibility of the synthesis of CuC_12_ through ball milling and the possibility of reducing the solvent content through this methodology, drawing the following conclusions:

From the crystal and molecular characterization, it can be concluded that the desired material, CuC_12_, is obtained through ball milling. In the X-ray diffraction analysis, proper purity of the sample was detected (>95 wt.%), with a minimal content of impurities (<5 wt.%) of the unreacted copper salt raw material and non-ionically linked dodecylamine hydrochloride.

The thermal performance of the BM samples presents values similar to those obtained by traditional methods, showing that the effect of impurities on the material’s thermal performance is minimal and highlighting the potential of ball milling to obtain suitable thermal energy storage materials.

The thermogravimetric analysis observed an additional decomposition step attributed to the unreacted dodecylamine hydrochloride.

The sustainability assessment elucidates the reaction and environmental efficiency of the synthesis through ball milling, where all the BM samples present similar sustainable results.

The reduction in the solvent content through ball milling did not significantly alter the structure or properties of the material.

Overall, the study validates the viability of obtaining CuC_12_ through ball milling and highlights its environmental benefits compared to traditional synthesis methods. Furthermore, ball milling allowed the solvent content of the synthesis to be reduced, further improving the sustainability of the process. Additionally, the reduction of solvent content in the experiments did not show a clear impact on the synthesis, although several differences were noted regarding the purity of the obtained material. This is a preliminary work; hence, future work should validate this procedure with different bis­(alkylammonium) tetrachlorometallates and assess ball milling as a solventless synthesis method.

## Supplementary Material



## Notes

X-ray diffraction analysis immediately after the milling process; sustainability evaluation, including real atom economy, environmental factor, process mass intensity, EcoScale, and energy consumption with corresponding equations and results (PDF)
