# Supplementary material for: Synthesis of Bis(dodecylammonium) Tetrachlorocuprate Using Ball Milling for Thermal Energy Storage
Source: ACS Sustain Chem Eng. 2025 Mar 26;13(13):4937–45. doi: 10.1021/acssuschemeng.4c08677 (PMC12164809; doi:10.1021/acssuschemeng.4c08677)
Supplement: Supplementary file 1 [file sc4c08677_si_001.pdf]

Supporting Information

Synthesis of bis(dodecylammonium) tetrachlorocuprate using ball milling for thermal energy storage

Rebeca Salgado-Pizarro, Jofre Mañosa, Camila Barreneche, Ana Inés Fernández*

Departament de Ciència de Materials i Química Física, Secció de Ciència de Materials, Facultat de Química, Universitat de Barcelona, C/ Martí i Franquès 1-11, 08028, Barcelona, Spain

*Corresponding author: c.barreneche@ub.edu

Supporting Information consists of 1 figure, 2 tables and 4 equations over 2 pages.

X-ray diffraction analysis immediately after the milling process

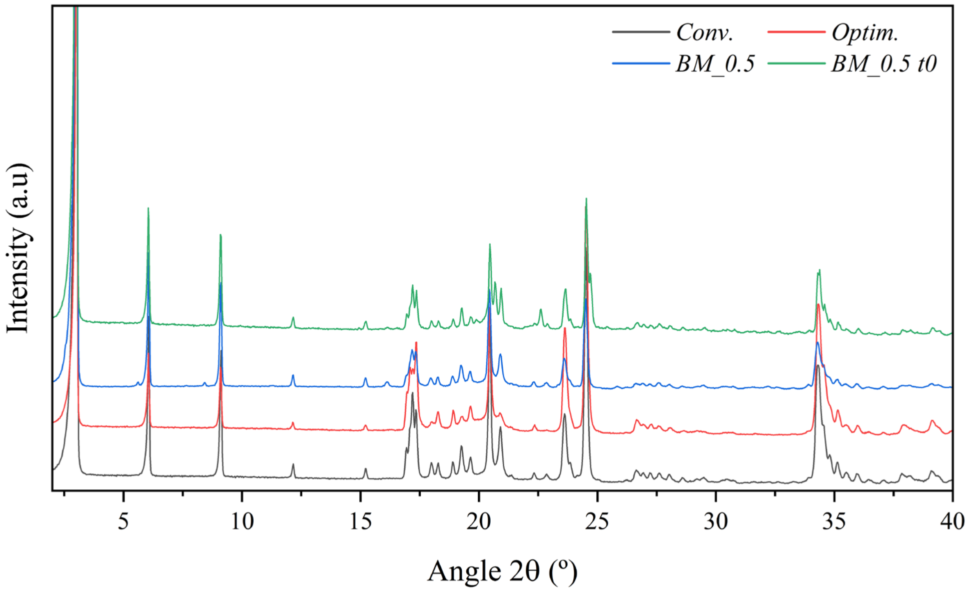

Figure S1 XRD diffractograms of the BM_0.5, immediately after the ball milling, and compared to traditional synthesis methods.

Sustainability evaluation, equations and calculations

S.1 Real Atom Economy (RAE)

$$RAE = \frac{real\ weight\ product\ (g)}{total\ weight\ of\ all\ the\ reactants\ in\ the\ process\ (g)} \cdot 100 \qquad \text{Eq. S1}$$

S.2 Environmental factor (E-Factor)

$$E - Factor = \frac{mass\ of\ wastes\ (g)}{mass\ of\ product\ (g)} \qquad \text{Eq. S2}$$

S.3 Process mass intensity (PMI)

$$PIM = \frac{total\ mass\ used\ in\ the\ process\ (g)}{mass\ of\ product\ (g)} \qquad \text{Eq. S3}$$

S.4 EcoScale

The following Table S1 explains our cases' penalty points and EcoScale scores.

Table S1 EcoScale scores.

Parameter \ Penalty points	Conv.	Optim.	BM_7.6	BM_5.7	BM_3.8	BM_2.0	BM_1.0	BM_0.5
**1. Yield** (100 – %yield)/2	16.86	5.86	8.02	1.26	9.10	7.56	2.43	7.82
**2. Price of reaction components** *(to obtain 10 mmol of product)*								
Dodecylamine – \$ 0.50 < \$10	0	0	0	0	0	0	0	0
HCl – \$ 0.05 < \$10	0	0	0	0	0	0	0	0
$CuCl_2 \cdot 2H_2O$ – \$ 0.16 < \$10	0	0	0	0	0	0	0	0
Methanol – \$ 4,26 < \$10	0	0	0	0	0	0	0	0
**3. Safety**								
Dodecylamine – N, T+	15	15	15	15	15	15	15	15
HCl – T	5	5	5	5	5	5	5	5
$CuCl_2 \cdot 2H_2O$ – N, T	10	10	10	10	10	10	10	10
Methanol – T, F	10	10	10	10	10	10	10	10
**4. Technical setup**								
Common setup	0	0	0	0	0	0	0	0
Instruments for controlled addition of chemicals	1	1	0	0	0	0	0	0
**5. Temperature/time**								
Heating > 1 h	3	3	0	0	0	0	0	0
Room temperature, < 24 h	1	1	1	1	1	1	1	1
**6. Workup and purification**								
Crystallisation and filtration	3	0	0	0	0	0	0	0
**Total penalty points**	65	51	49	42	50	49	43	49
**EcoSacale value (%)**	35	49	51	58	50	51	57	51

S.5 *Energy Consumption*

Energy consumption is calculated using the Eq. S4.

$$Energy\ consumption\ (J \cdot g^{-1}) = \frac{Energy\ consumed\ (Ws)}{mass\ of\ product\ (g)} \qquad \textit{Eq. S 4}$$

The mass of the product obtained was standardised since the theoretical mass of the *Conv.* and *Optim.* methods was 5.8 g and the *BM* 9.56 g. Considering that in the *Conv.* method, the final mass of the product is after the recrystallisation process, and the *Optim.* and *BM* are not. The final mass of the *BM* was recalculated using the experiment yield with a theoretical mass of 5.8 g.

S.6 *Summary of the calculations*

Table S2 Results of all the sustainable key indicators analysed.

Data & KPIs \ Synthesis Method	*Conv.*	*Optim.*	BM_7.6	BM_5.7	BM_3.8	BM_2.0	BM_1.0	BM_0.5
Yield (%)	66.28	88.28	84.79	97.49	81.80	84.88	95.15	84.35
Dodecylamine (g)	3.7	3.7	6.13	6.13	6.13	6.13	6.13	6.13
Hydrochloric acid (g)	2.28	2.28	3.57	3.57	3.57	3.57	3.57	3.57
Copper chloride dihydrated (g)	1.7	1.7	3.04	3.04	3.04	3.04	3.04	3.04
Methanol (g)	75.1	39.5	67.2	53.3	39.5	26.4	19.1	15.5
Bis(dodecylamine) tetraclorocuprate (g)	1.801	5.26	8.11	9.32	7.82	8.11	9.10	8.06
Real Atom Economy (%)	2%	11%	10%	14%	15%	21%	29%	29%
Environmental factor	41.67	7.51	8.28	5.72	5.05	3.25	2.10	1.92
Process mass intensity	45.68	8.89	9.86	7.09	6.68	4.82	3.50	3.50
EcoScale (%)	35	49	51	58	50	51	57	51
Energy consumption ($MJ \cdot kg^{-1}$)	937.48	193.63	28.49	24.78	29.54	28.47	25.39	28.64
